# Prevalence and severity of pediatric cases in Stockholm’s physician-staffed prehospital units: a retrospective cohort study

**DOI:** 10.1186/s12873-024-01126-3

**Published:** 2024-11-12

**Authors:** Denise Bäckström, Henrik Jörnvall, Erik Strandqvist, Robert Ahlerup, Rebecka Rubenson Wahlin

**Affiliations:** 1 Department of Biomedical and Clinical Sciences, Linköping University, Linköping, 581 83 Sweden; 2 Capio Akutläkarbilar, Stockholm, Sweden; 3 Department of Physiology and Pharmacology Section for Anesthesia and Intensive Care Medicine, Karolinska Institutet, Stockholm, Sweden; 4 Function Perioperative Medicine and Intensive Care, Department of Perioperative Care, Solna Karolinska University Hospital, Stockholm, Sweden; 5 Department of Pediatric Perioperative Medicine and Intensive Care, Astrid Lindgren Children’s Hospital, Karolinska University Hospital, Stockholm, Sweden; 6 Peri-Operative Medicine & Intensive Care Function, Peri-Operative Medicine, Karolinska University Hospital, Huddinge, Sweden; 7 Division of Anaesthesia and Intensive Care, Department of Clinical Science, Intervention and Technology, Karolinska Institutet, Stockholm, Sweden

**Keywords:** Prehospital, Rapid response vehicle, Scandinavia, Sweden, Pediatric, Children, P-EMS

## Abstract

**Background:**

Ambulance missions involving pediatric patients are common in emergency medical services (EMS) globally, with variations in prevalence based on geographic location.

This retrospective cohort study analyzes the prehospital physician staffed units (p-EMS) in Stockholm, assignment dispatches and the prehospital characteristics and interventions involved, from January 1, 2021, to December 31, 2022.

**Methods:**

Utilizing data from LogEze, a quality assurance system, we reviewed all Rapid Response Vehicle (RRV) operations in the Stockholm Region, which totaled 4,682 pediatric assignments. The analysis included dispatch types and interventions assessing the frequency and nature of pediatric prehospital missions.

**Results:**

Pediatric cases constituted 20.0 % of RRV dispatches, with the majority involving respiratory distress, seizures, and blunt trauma. Despite high dispatch rates, advanced medical interventions were seldom required, indicating most pediatric cases were not severe. Stand-downs occurred in 30.1 % of cases, reflecting the cautious approach in pediatric dispatches. Furthermore, the study observed a significant reliance on p-EMS for complex pediatric cases, underscoring the value of specialized training and resources in managing such emergencies.

**Conclusion:**

The study highlights the crucial role of p-EMS in enhancing pediatric emergency care in Stockholm. Despite frequent pediatric dispatches, the low incidence of severe cases underscores the need for precise triage and resource allocation. This analysis supports the need for continuous training and resource optimization in p-EMS to ensure high-quality care for pediatric patients across varied emergency scenarios.

**Supplementary Information:**

The online version contains supplementary material available at 10.1186/s12873-024-01126-3.

## Background

 Ambulance missions involving pediatric patients are a frequent occurrence in emergency medical services (EMS) worldwide [1, 2]. The prevalence of such cases varies based on geographic location. In Scandinavia 4.5–6.9% of ambulance missions involve children under 16 [3, 4]. A lower socioeconomic status of a neighbourhood is associated with a higher need for EMS dispatch for children [5].

Prehospital care of children constitutes several challenges. A particular concern is that the EMS workers typically does not have specific training involving acute care of children. Since there are less children involved in the acute EMS care the EMS staff tends to care for a smaller number of young patients per worker [2]. The impact of providers’ discomfort with the patient’s age on medication problems, equipment size and parental interference significantly influences adverse events and near misses in paediatric care settings [6].

Ambulance services face distinct challenges when providing care to pediatric patients. Children have unique physiological and psychological needs that necessitate specialized attention. The assessment and treatment of pediatric patients require healthcare providers to adapt their approach to the child’s age, weight, and developmental stage, as well as to consider their inability to provide a comprehensive medical history [7, 8]. There is, among other factors, a tendency for vital parameters to be less commonly documented for younger children [9, 10]. The provision of pain management, can be particularly challenging [11]. Managing the emotional distress of both the child and their caregivers is essential in ensuring effective care delivery [12].

The prevalence of pediatric missions within prehospital physician-staffed units (p-EMS) displays a considerable degree of variability and has been reported to be 1.5–27% [13–17]. This observed heterogeneity in the incidence of pediatric cases highlights the complex and multifactorial nature of emergency medical services. The disparities in pediatric mission rates may stem from diverse factors, including regional demographics, geographical factors, healthcare system organization, and population health dynamics. Understanding and addressing these differences in pediatric mission prevalence are crucial for optimizing resource allocation, training, and preparedness of medical teams to ensure that pediatric patients receive high-quality, tailored care, regardless of their geographic location.

Prehospital physician-staffed units can make a significant difference in addressing the challenges EMS faces in providing care for children. Common procedures performed by p-EMS include oxygen supplementation, intravenous access and application of a cervical collar application, although uncommon tracheal intubation, intraosseous access and chest drainage are also procedures provided by p-EMS [14, 18, 19].

Understanding the frequency and nature of pediatric prehospital missions is critical for optimizing resource allocation, training, and preparedness within the EMS system, ensuring that the unique medical needs of children are met with the highest standard of care [2, 20]. Further investigation and analysis of these missions can aid in developing targeted interventions to improve the quality and efficiency of pediatric prehospital care.

To our knowledge no study has explored the types of dispatches and interventions performed by p-EMS in Sweden. This study aims to describe the prehospital care of children by investigating the p-EMS units in Stockholm assignment dispatches and the prehospital characteristics and interventions involved.

## Method

### Study design and participants

This retrospective cohort study was based on data from LogEze, a quality assurance system where all assignments are registered. The study includes data for all RRVs operating in the Stockholm Region from January 1, 2021, to December 31, 2022.

### Setting

Stockholm has an area of 6,519 km^2^ and a population of approximately 2.4 million. In 2021, the total number of children aged 0–17 was 520,121, and by November 1st, 2022, it had decreased slightly to 518,943.

This region is served by seven emergency departments, with major trauma cases directed to the regional trauma center. When it comes to transporting children, the Emergency Medical Services (EMS) have designated three hospitals: Nya Karolinska Hospital in Solna, Karolinska Hospital in Huddinge, and Sachsska Children’s Hospital. Dispatching of RRVs occurs from the dispatch center based on identified or suspected illnesses. At the dispatch center, the staff primarily consists of non-medically trained personnel who follow predefined templates and guidelines for activating prehospital units in. There are also some nurses and a physician present; however, they are not involved in all dispatches.

EMS crews can also request RRVs for medical assistance while attending to a patient. Within the EMS, there are three primary categories of healthcare workers, (1) Prehospital emergency nurses, professionals that holds a nursing degree along with a graduate degree in prehospital emergency care; (2) ambulance nurses, holding a nursing degree and provide essential medical care in the prehospital setting, and (3) Emergency Medical Technicians, Their medical training varies from six months to two years, equipping them to deliver critical care in emergency situations. The extent of pediatric training varies. All nurses receive pediatric education during their three-year undergraduate program, and the majority have also completed specific courses, such as “Pediatric Education for Prehospital Professionals” or similar training.

In Stockholm during the study period, there were two daytime RRVs staffed with a nurse anesthetist and an emergency physician, and one vehicle that operated around the clock staffed with an anesthesiologist and a prehospital emergency nurse. All three vehicles handled pediatric cases, and there was no distinction in the type of assignments allocated to the different vehicles; the nearest available vehicle was assigned the task by the dispatch centre.

The dispatch of RRV occurs through two primary mechanisms: the dispatch center follows a predefined set of criteria or an on-site ambulance requests support from a physician-staffed emergency vehicle. Criteria for dispatching RRVs include suspected compromised airways, acute missions, support and assistance to other prehospital units, patient assessments to determine the necessary level of care, and handling on-site deaths if an on-call family physician cannot arrive promptly. The dispatch center uses a computerized system that automatically recommends deploying an RRV for all priority 1 cases involving children. However, deployment is not always possible, particularly at night when only one vehicle is operational and may be occupied with other duties.

### Bias

Rapid response vehicle (RRV) staff register every assignment directly at the site in the LogEze quality assurance system, using a smartphone, tablet, or computer. There is no mandatory information requirement or time limit for registration. This study is based on the information in the quality assurance system. Information documented is the perceived situation by the RRV, such as the NACA score, it is not based on the patient’s diagnosis and subsequent hospital care.

### Variables

#### Priority levels

Priority 1 indicates a very urgent assignment for a life-threatening condition. Priority 2 denotes an urgent assignment for an acute, yet non-life-threatening condition. Priority 3 signifies a non-urgent assignment for a non-acute condition, with no adverse impact on the patient from waiting. Priorities 4–9 suggest that the patient requires assessment for a non-urgent or non-life-threatening condition. Priorities 5 and 9 typically involve a visit from a nurse or family doctor [21].

#### Stand-downs

If ambulance personnel decide that they do not need assistance from an RRV, they make radio contact to report the patient’s SBAR (Situation, Background, Assessment, Recommendation). Following the ambulance’s report that no further assistance is required, the RRV physician decides whether to abort the assignment or proceed.

For additional variables, see Appendix 1.

### Statistics

The data were analysed using Microsoft Excel 365 (Microsoft Corp, Redmond, WA, USA). Data were presented with numbers and percentage. Data imputation was not used to correct for missing values. Population data for calculating incidence were obtained from Statistics Sweden [22].

## Results

During 2021 and 2022, RRVs in Stockholm were dispatched on 23,464 occasions, of which 4,682 (20.0%) where identified as pediatric patients aged 0 to 17 years of age and that was analysed further in the present study. Of these, 17 are duplicate registrations, indicating that more than one RRV recorded the mission. This occurs when an RRV closer to the patient becomes available, allowing for a switch of vehicles. The five most common dispatch categories were respiratory distress 850 (18.2%), seizures 837 (17.9%), blunt trauma 798 (17.0%), anaphylactic reaction 458 (9.8%), and depressed level of consciousness 361 (7.7%).

**Fig. 1:**
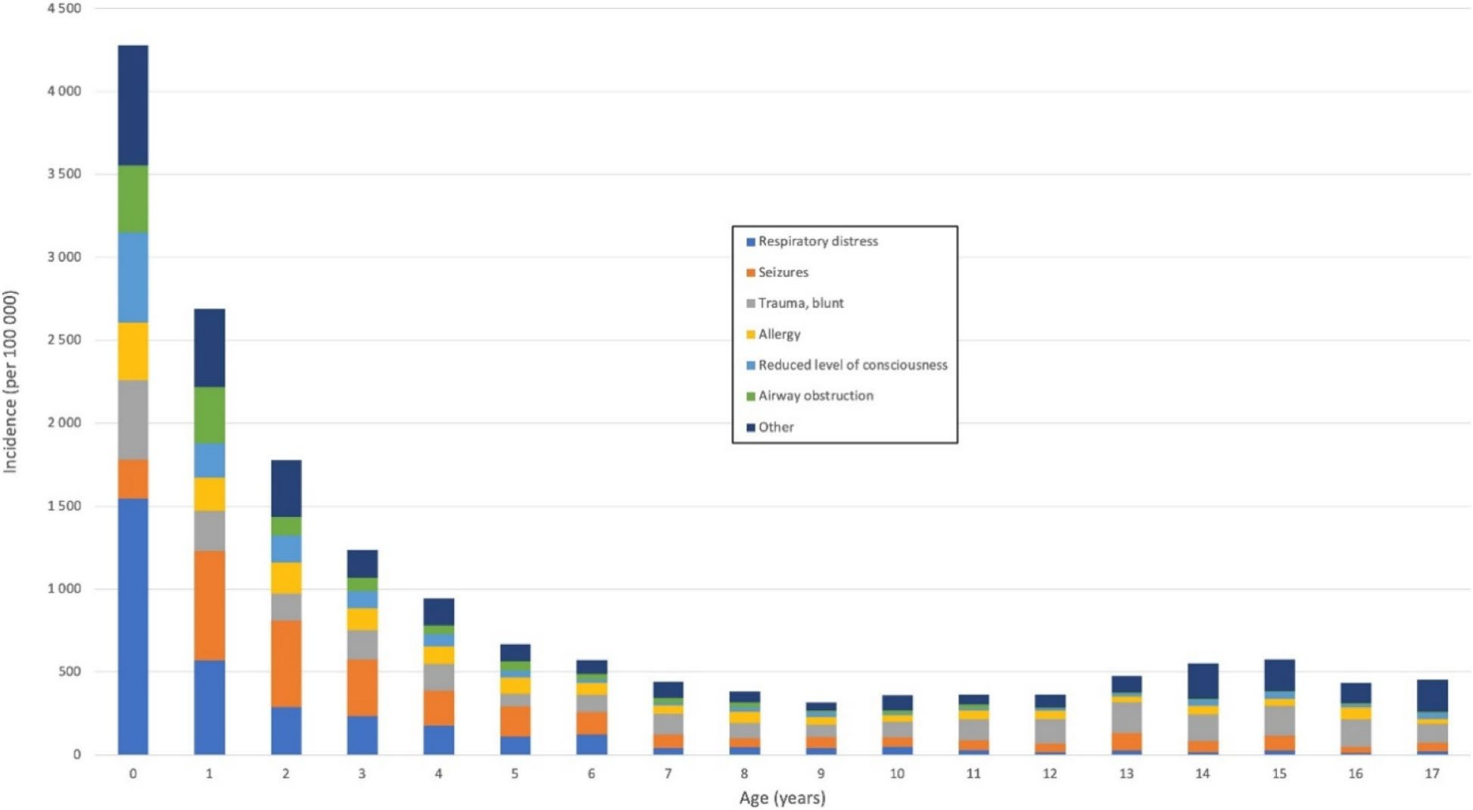
Incidence per 100,000 in each age (years) in children

**Fig. 2:**
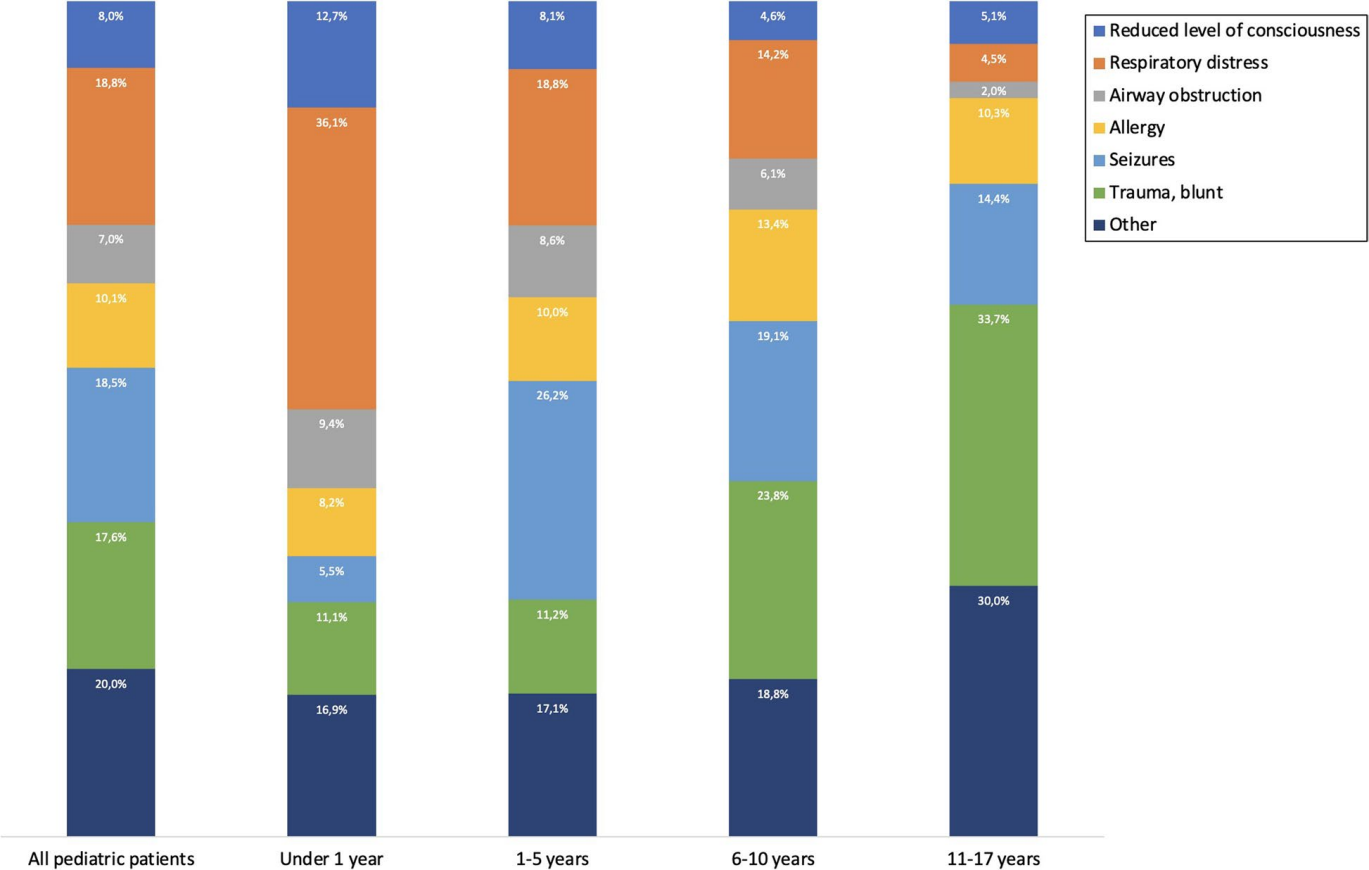
Dispatch categories in age groups

The majority of the missions concerned patients under 6 years of age, with 982 (21.0%) under the age of 1 year and 2,099 (44.8%) of age 1 to 5 years. The relative incidence of the dispatch categories varied greatly when the missions were portioned by age (Fig. 1). Respiratory distress was the most common dispatch category in missions to patients under 1 year of age (1,546 per 100,000 children-years), and the incidence then dropping through preschool ages to becoming very rare after year 6. Other common dispatch categories in patients during their first year was altered consciousness (544 per 100,000 children-years), blunt trauma (476 per 100,000 children-years), airway obstruction (403 per 100,000 children-years) and allergy (349 per 100,000 children-years). Seizures were relatively uncommon (236 per 100,000 children-years) among patients less than 1 year of age (Fig. 2).

**Fig. 3:**
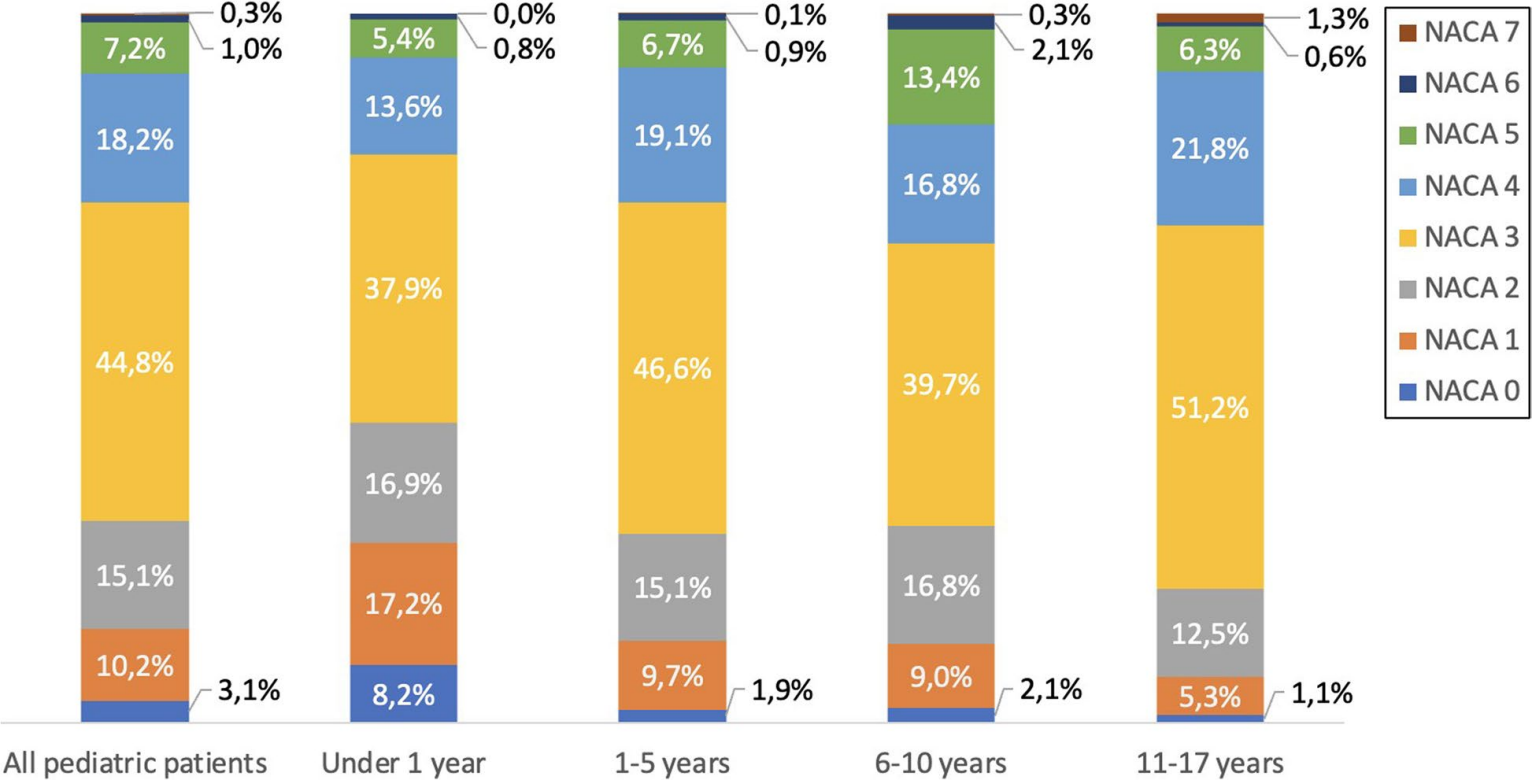
NACA scores in age groups

However, the NACA score also differed between the different dispatch categories (Fig. 3). Thus, the number of patients with a NACA score > 3 in patients less than 1 year of age and in the different categories were 54 of 341 (15.8%) for respiratory distress, 11 of 120 (9.2%) for altered consciousness, 4 of 105 (3.8%) for blunt trauma, 10 of 89 (11.2%) for airway obstruction, 12 of 77 (15.6%) for allergy, and 10 of 52 (19.2%) for seizures. Blunt trauma was a common dispatch category for all ages, and as the patients became older, the dispatch category became more heterogeneous.

In total, 1,409 (30.1 %) cases resulted in stand-downs. In 522 cases (11.1 %), the RRV team reported that they had a significant impact on the healthcare provided, or that they deviated from current guidelines or standard operating procedures based on the condition of the individual patient.

The dispatch priority in paediatric cases was almost exclusively (4,532, 97.5 %) priority 1. NACA score was registered in only 2880 cases, the most common NACA score was III (1,291, 44.8%), followed by NACA IV (524, 18.2%), NACA II (436, 15.1%), NACA I (295, 10.2%), NACA V (208, 7.2%), NACA 0 (no injury or disease; 88, 3.1%), NAVA VI (28, 1.0%) and NACA VII (10, 0.3%). Combined, NACA 0-III with non-threatened vital parameters encompassed 2,110 (73.3%) of the cases, with remaining 770 (26.7 %) being NACA IV-VII (Fig. 3).

The NACA score were further analysed in different age groups: under 1 year, 1–5 years, 6–10 years, and 11–17 years. In patients under 1 year, 489 of 610 (80.2%) had a NACA score of 3 or less, while the corresponding fraction among 11–17 years old patients were 448 of 639 (70.1%). Remarkedly, 50 (8.2%) of the patients under 1 year had a NACA score of 0, denoting no injury or disease. Notable, there was no patient under 1 year with NACA VII, probably reflecting the fact that irrespectively of the perceived condition or prognosis, CPR is conducted continuously, and the patient always brought to the emergency department in cases of cardiac arrest in infants.

Specific interventions, procedures or unique medications only provided by the RRVs was uncommon among the paediatric cases, all below 1% of the cases with the only exception of advanced pain medication in 54 cases (1.2%). However, 161 (3.4%) of the dispatches originated in a request from the ambulance first at scene, reflecting the relative importance of a physician staffed assisting unit. The physician or nurse in the RRV assisted with conveyance to hospital in 560 (12.0%) cases, the majority of them in ground ambulances 517 (92.3%) and only 43 (7.7%) of them in helicopter ambulances.

## Discussion

Our dispatch rate of 20.0 % pediatric cases within p-EMS is a considerable proportion, meriting scientific scrutiny and consideration. It is exceeding the reported rates of most p-EMS [13, 14, 16, 17], although there are some exceptions where a higher proportion has been documented [15]. Pediatric cases often present unique clinical challenges and require tailored medical interventions, necessitating the deployment of appropriately trained medical personnel and dedicated resources.

We have only found one study reporting a higher proportion of pediatric cases, describing the Rotterdam physician-based HEMS missions [15]. Oude Alink et al. attribute the high volume of missions to a deliberate strategy of overtriage. Our data suggest that a similar approach exists in Stockholm, although it is not explicitly stated. Sweden exhibits one of the world’s lowest child mortality rates attributed to injuries [23]. The exceptionally low incidence of child fatalities resulting from injuries can be attributed to a multifaceted approach, which encompasses strict safety regulations, well-established child protection measures, and comprehensive public health initiatives [24]. The observed disparities in our study are unlikely to be attributed to a higher prevalence of severely injured or ill children in Sweden, but rather, are likely to stem from variations in the dispatch criteria and, potentially, the level of familiarity among ambulance personnel in managing pediatric missions.

Our study revealed that respiratory distress 18.2 % and seizures 17.9 % were the most frequently observed chief complaints among pediatric patients. These findings are consistent with prior research conducted in EMS [1, 3, 17, 25, 26], indicating a recurring pattern of presenting complaints in pediatric prehospital care. The high prevalence of respiratory distress is indicative of the significance of pulmonary conditions among pediatric patients, which may encompass a wide range of respiratory issues. Similarly, seizures are a common and concerning presentation in the pediatric population, which might require immediate attention and intervention.

In our study, a substantial proportion of pediatric cases, approximately 17.0 %, presented with blunt trauma as their primary condition. This finding highlight trauma as a leading cause of injury and morbidity among children [27]. Blunt trauma encompasses a wide spectrum of mechanisms, including falls, motor vehicle accidents, sports-related injuries, and physical assaults, among others and is a known reason for dispatch of EMS [1, 3, 25, 28]. The high frequency of blunt trauma among pediatric patients is reflective of the inherent vulnerability of children to external forces and accidents, which may result in various types of injuries, ranging from minor to severe [29].

Cases classified as NACA scores 0 to III, where vital parameters were not indicative of immediate life-threatening conditions, constituted the majority, accounting for 73.3 % of the total cases. In contrast, the remaining 26.7% of cases fell into the NACA IV-VII range, indicating more severe and potentially life-threatening conditions. Similar NACA values have been observed in a study from Germany, indicating comparable clinical acuity levels in the patient population [14]. The prevalence of severe illness or injury as defined by a NACA score of at least 4 varied greatly among the dispatch criteria. Given the abundance of patients under the age of 1 year (21.0% of all missions), it is important to address any potential over triage among these patients. In this respect it is interesting that only 3.8 % of the trauma patients in this age group had a NACA score > 3, while 19.2% among the patients with seizures. Other dispatch criteria showed a similar pattern of over triage (altered consciousness 9.2%, respiratory distress 15.8%, airway obstruction 11.2%, and allergy 15.6%). Thus, focus to improve the dispatch procedure should be made in cases of blunt trauma and to some degree also altered consciousness, potentially saving large resources better needed elsewhere. Given that the personnel at the dispatch center do not possess medical training and merely follow guidelines, we do not perceive this as a lack of specific pediatric knowledge among the dispatch center staff. Their knowledge is consistent for both children and adults. Thus, we believe their guidelines need to be revised.

A substantial proportion of cases, approximately 30 %, resulted in stand-downs, it is noteworthy that the rate of stand-downs for pediatric patients remained somewhat lower than that observed for adult patients during the same period [30]. This discrepancy in the frequency of stand-downs between the two patient populations marks the differential considerations and assessment criteria applied by emergency medical services in response to pediatric versus adult cases. The lower incidence of stand-downs for pediatric patients may be indicative of a greater caution and attention to ensuring the timely dispatch of resources to pediatric emergencies, even in cases where the situation may ultimately be less critical than initially perceived. It emphasizes the dedication to pediatric care within the emergency medical services system, prioritizing the well-being of pediatric patients even in instances where the situation may not warrant immediate attention.

In our study, we identified relatively low utilization rates of advanced medical interventions for pediatric patients. The administration of advanced pain medications was observed in only 1.2% of cases, while the application of assisted breathing and intubation were reported in 1.8% and 0.9% of cases, respectively. These findings suggest a low population of severely ill and injured children as well as cautious and selective approach to the use of these advanced medical interventions in the prehospital care of pediatric patients [31].

The number of intubated children in our study (0.9%) is low compared to other p-(H)EMS units that range from 3.7 to 18.8% [14–16, 32]. Prehospital anaesthesia and intubation of pediatric patients are critical procedures that carries both significant importance and inherent risks [33]. While intubation is a key intervention to secure the airway and ensure adequate oxygenation and ventilation in certain critical cases, it is accompanied by specific considerations and potential challenges when applied to children. Pre-hospital intubation of children can be difficult and is considered an uncommon procedure [16, 34]. There is a significant difference in the difficulty of pre-hospital intubation between children and adults [35]. In each RRV in Stockholm, there is a physician or nurse specialized in anesthesia who is experienced in airway management, including in children. The low number of intubations may not only indicate a lack of patients with difficult airways but could also suggest that the staff felt confident using alternative methods.

A notable observation from this study is that 3.4 % of pediatric missions were initiated at the request of the ambulance personnel seeking assistance from the p-EMS, similar numbers have been seen in the Netherlands [15]. This finding stresses the significance of interprofessional collaboration in prehospital healthcare, particularly when dealing with pediatric cases. Collaboration between p-EMS and other professional emergency services is common, especially EMS [36]. The fact that ambulance personnel proactively sought medical expertise from the physician-staffed unit in a number of cases indicates the complexity and critical nature of some pediatric emergencies. Overall, this finding sheds light on the valuable role that physician-staffed units play in supporting ambulance personnel when confronted with complex pediatric cases, ultimately enhancing the quality of care provided to these young patients.

In 12% of the dispatches, the physician accompanied the patient to the hospital. This additional support from a physician during transport occurred primarily with ground ambulance units and only with a smaller proportion, 7.7%, involving transport via helicopter. These findings illuminate the adaptability and responsiveness of prehospital healthcare systems in Sweden, wherein physicians can be dynamically deployed to accompany patients in accordance with their clinical needs.

The choice between ground ambulance and helicopter transport as the mode of transportation is influenced by a multitude of factors. Critically ill or unstable patients may benefit from the speed and advanced medical capabilities of helicopters, which offer a controlled and expedited environment for patient care [37]. Patients with less severe conditions or those in stable health may be better suited for ground ambulance transport, which provides a more cost-effective and efficient solution. These variations in the choice of ground ambulance or helicopter transport may account for the differing distributions observed across different locations, such as the reported 26.7% of missions involving helicopter transport in Germany [14]. Sweden, being a northern country, often experiences winter weather conditions that can render helicopter flights impossible. Stockholm, as a major city, faces traffic congestion at certain times of the day, making helicopter transport more advantageous than ground ambulances. Ultimately, the choice of transportation method is governed by the patient’s medical needs, though various other factors also play a role in the relatively low utilization of helicopter transports observed in our study.

Retrospective data collection and the utilization of forms that do not mandate the completion of all required information do possess inherent limitations. These limitations primarily revolve around the potential for incomplete or missing data, which can hinder the accuracy and comprehensiveness of the recorded information. It is worth noting that these shortcomings have been partially mitigated by the advantage of data accessibility by both team members. This cooperative effort fosters a more thorough and precise documentation process, addressing some of the limitations associated with retrospective data collection and incomplete forms.

The study was conducted within a specific region of Sweden; however, its findings bear broader significance for all professionals involved in prehospital healthcare. The findings emphasize that the care of pediatric patients requires a distinct skill set and experience due to the limited cases. These findings have the potential to inform and influence prehospital healthcare providers across various locations, as they highlight the imperative of adequate training, standardized protocols, and specialized resources for the effective management of pediatric cases.

## Conclusion

Our findings reveal that dispatches for pediatric missions are relatively common in Sweden, reflecting the recognition of pediatric emergencies within the prehospital setting. However, it is noteworthy that the majority of these dispatched pediatric cases do not present with severe illness or injury. In fact, interventions in these scenarios are infrequent. This discrepancy between the frequency of dispatches and the necessity for critical interventions underscores the importance of maintaining and enhancing the competence of physician-staffed prehospital teams in pediatric care and also highlights the significance of a proficient dispatch service.

## Supplementary Information

Supplementary Material 1.

## Data Availability

The ethical approval stipulates that data generated and/or analysed during this study are not publicly available and can only be distributed to the research group. However, reasonable requests for further information can be addressed to the corresponding author.

## Abbreviations

EMS: Emergency Medical Services
p-EMS: Physician-staffed Emergency Medical Services
RRV: Rapid Response Vehicle
NACA: National Advisory Committee for Aeronautics Score
SBAR: Situation, Background, Assessment, Recommendation
