# Supplementary material for: Prevalence and severity of pediatric cases in Stockholm’s physician-staffed prehospital units: a retrospective cohort study
Source: BMC Emerg Med. 2024 Nov 12;24:211. doi: 10.1186/s12873-024-01126-3 (PMC11555878; doi:10.1186/s12873-024-01126-3)
Supplement: Supplementary file 1 — Supplementary Material 1. [file 12873_2024_1126_MOESM1_ESM.docx]

Appendix 1

**Variables**

**Age**

in days, months and years

**Dispatch category**

Registration includes operational data for the following variables: Cardiac Arrest, Cardiology (N/S), Unconsciousness, Decreased Level of Consciousness (LOC), Shortness of Breath, Airway Obstruction, Allergies, Seizures, Neuro/Stroke, Blunt Trauma, Penetrating Trauma, Burns, Intoxication, Drowning, Psychiatry, Obstetrics, Medical (N/S), Surgical (N/S), Infection (N/S), Suspected Healthcare Needs, Active Shooting, and Other.

Injured body regions include the head, torso, and extremities.

Types of trauma encompass traffic injuries, falls, assaults, hangings, drownings, smoke inhalation, hypothermia, and other.

Weapons used include knives, guns, and other.

**Priority level**

Each patient is assigned a National Advisory Committee for Aeronautics (NACA) rating as follows: NACA 0: no injury/disease; NACA I: minor injury/disease – no treatment needed; NACA II: injury/disease requires examination but not hospital admission; NACA III: injury/disease without acute threat to life but requiring hospital admission; NACA IV: injuries/diseases possibly leading to deterioration of vital signs; NACA V: injuries/diseases with acute threat to life; NACA VI: requires CPR; NACA VII: patient deceased.

**Subjective impact of healthcare provided**

The RRV team also has the ability to document whether they perceive that they had a significant impact on the care provided to the patient or if they deviated from the established guidelines.

**Interventions, procedures and medications provided**

Interventions performed by RRV include the following: rapid sequence induction (RSI), intubation without any sedative medication, vasopressor during cardiac arrest, vasopressor other, advanced pain relief (dosage other than ambulance guidelines). Intubation without medication can be performed by some prehospital nurses (i.e. trained anaesthesia nurses). RSI can only be performed by RRVs and ambulance helicopters in Stockholm.

RRVs carry specialized equipment not found in regular ambulances, including a video laryngoscope (MacGrath), ultrasound (Butterfly), and a mechanical chest compression device (LUCAS). RRVs also carry medications not found in regular ambulances, including magnesium, oxytocin, hydrocortisone, flumazenil, tranexamic acid, sodium chloride, calcium gluconate, and hydroxocobalamin.

**First on scene**

The RRV team may also record if they arrive first on scene. This means that the assigned ambulance for the specific assignment arrives after the RRV.

**Conveyance to hospital**

If the Rapid Response Vehicle (RRV) team identifies a need to accompany the patient to the hospital, or if the ambulance crew requests their presence, either the doctor or the nurse from the RRV can fulfill this role. This may also be a request originating from the ambulance crew.

**Method of transportation**

Registration can document how the team arrived at the scene, whether by car or helicopter, and also include the method of patient transport to the hospital.
